# Selective Ammonia-Sensing Platforms Based on a Solution-Processed Film of Poly(3-Hexylthiophene) and p-Doping Tris(Pentafluorophenyl)Borane

**DOI:** 10.3390/polym12010128

**Published:** 2020-01-05

**Authors:** Alem Araya Meresa, Felix Sunjoo Kim

**Affiliations:** School of Chemical Engineering and Materials Science, Chung-Ang University, Seoul 06974, Korea; alemaraya12man@gmail.com

**Keywords:** polymer blend, tris(pentafluorophenyl)borane, poly(3-hexylthiophene), organic field-effect transistor, chemical sensor, Lewis acid, doping and dedoping

## Abstract

Here, we fabricate ammonia sensors based on organic transistors by using poly(3-hexylthiophene) (P3HT) blended with tris(pentafluorophenyl)borane (TPFB) as an active layer. As TPFB is an efficient p-type dopant for P3HT, the current level of the blend films can be easily modulated by controlling the blend ratio. The devices exhibit significantly increased on-state and off-state current levels owing to the ohmic current originated from the large number of charge carriers when the active polymer layer contains TPFB with concentrations up to 20 wt % (P3HT:TPFB = 8:2). The current is decreased at 40 wt % of TPFB (P3HT:TPFB = 6:4). The P3HT:TPFB blend with a weight ratio of 9:1 exhibits the highest sensing performances for various concentrations of ammonia. The device exhibits an increased percentage current response compared to that of a pristine P3HT device. The current response of the P3HT:TPFB (9:1) device at 100 ppm of ammonia is as high as 65.8%, 3.2 times that of the pristine P3HT (20.3%). Furthermore, the sensor based on the blend exhibits a remarkable selectivity to ammonia with respect to acetone, methanol, and dichloromethane, owing to the strong interaction between the Lewis acid (TPFB) and Lewis base (ammonia).

## 1. Introduction

The fabrication of chemical detectors has attracted considerable attention owing to their applications in health and environmental monitoring [1,2,3,4,5,6]. Among the various analytes, ammonia is of interest owing to its presence in industrial, agricultural, biomedical, and living environments. The concentration of ammonia should be maintained below the threshold concentration [7,8,9,10,11]. The limit value of ammonia exposure is only 25 parts per million (ppm) for long-term exposure (i.e., 8 h). Therefore, ammonia sensors are required to exhibit appropriate signals when the concentration exceeds the limit [7]. In terms of sensing capability, chemical sensors based on organic field-effect transistors (OFETs) are a very promising platform [1,2,3,4,5,6]. They directly transduce the chemical signal into an electrical signal, unlike other types [8,9,10,11,12,13,14,15]. Owing to the gate-induced signal amplification, OFET-based sensors are more scalable and sensitive than their resistor-based counterparts [16,17,18,19]. Diverse chemistry and functional groups of organic compounds can enable the design and fabrication of devices targeting specific analytes. OFETs can be operated at room temperature, which enables the use of the sensors in general environments. These characteristics of OFETs are also beneficial for sensing elements for physical and biological signals [5,6,18]. However, higher sensor performances accompanied by a scalable fabrication method are still required. 

The sensing mechanisms of chemical sensors are associated with the interactions between the device and analyte, which could affect the electrical characteristics of the OFETs through doping/dedoping of the organic semiconductors, trapping/quenching of charge carriers, alteration of the molecular arrangement of the active layer, or influence on the charge injection/extraction/transport at the various interfaces [8,19,20,21,22]. In other words, when the active sensing layer is exposed to external analytes, specific interactions occur between the active layer and analytes. Such interactions lead to the changes in charge-transport properties including the charge-carrier density and mobility, and then enable instant detection and response to the analyte [23,24]. For polar analytes, the charge transport in the organic semiconductor layer may also be disturbed by increasing the amount and magnitude of energetic disorders through charge–dipole interactions [25]. The quality, topography, and morphology of the active layer as well as the geometry of the devices can be optimized to enhance these properties [10,20,21,22]. 

There have been several reports regarding ammonia sensors based on polymer transistors. Noticeable materials for ammonia-sensing OFETs include various p-type polymers, such as poly(3-hexylthiophene) (P3HT), poly(2,5-bis(3-tetradecylthiophen-2-yl)thieno[3,2-b]thiophene) (PBTTT), poly(3,3′′′-didodecylquarterthiophene) (PQT-12), and poly[2,5-bis(7-decylnonadecyl)pyrrolo[3,4-c]pyrrole-1,4(2H,5H)-dione-(E)-1,2-bis(5-(thiophen-2-yl)selenophen-2-yl)ethene] (P-29-DPP-SVS) [9,10,11,12,13,22,25,26,27,28,29]. Surface morphologies of OFETs based on P3HT or PBTTT films have been tuned for enhanced ammonia-sensing capability [22,28]. OFETs based on single-crystalline nanowires of P3HT have showed a high sensitivity to ammonia at a concentration as low as 0.01 ppm [11]. A processing method compatible to roll-to-roll fabrication has been applied to OFET-based ammonia sensors on a plastic substrate with a detection limit of ~0.1 ppm [26]. Blending of P3HT with polystyrene (PS) has been reported as a promising strategy for ammonia gas sensing applications [27]. Such blends were effective in not only maintaining the OFET performance but also increasing the interfacial area to interact with analyte, resulting in the efficient detection. These OFET-based sensors have also exhibited a certain degree of selectivity to a specific analyte. 

The blending of a dopant, such as tris(pentafluorophenyl)borane (TPFB), with a p-type polymer poly(3-hexylthiophene) (P3HT), under certain circumstances, can be used to supply a large number of mobile charge carriers for a better electrical conductance and to improve the charge transport in organic semiconductors through the introduction of excess charge carriers which can in turn fill in unwanted trapping sites in the active semiconducting layer [30,31,32,33]. TPFB is a Lewis acid, which can act as an ammonia receptor in the active layer by complexation, owing to the strong Lewis acid–base interactions between the boron and nitrogen atoms [8]. Considering the high solubilities of P3HT and TPFB in common organic solvents, blend films can be easily deposited by a solution-based processing [30,31,32]. TPFB has been used for various organic electronic devices, including organic transistors and sensors [8,34,35,36]. 

In this study, we fabricated gas-phase ammonia sensors based on OFETs by using a blend of P3HT and TPFB as an active layer and compared their characteristics to those of pristine P3HT-based devices. Our focus was to enhance the sensor performance by utilizing an acid–base interaction between the active component and the analyte. The solubility of the components enabled a straightforward film formation by spin coating, which makes the system feasible for practical applications. We varied the weight ratio of P3HT and TPFB to study the changes in absorption spectra and OFET performances and observed characteristics of a doped polymer semiconductor in agreement with previous reports. We selected a P3HT:TPFB blend with a weight ratio of 9:1, as it provided a homogeneous film with reasonable electrical properties. In ammonia detection, the blend film exhibited a higher sensitivity, quantified by the relative current change, than that of the pristine P3HT film at the same film thickness and under the same test conditions. The P3HT:TPFB device not only exhibited a higher sensitivity but also remarkably increased selectivity with respect to other chemicals such as acetone, methanol, and dichloromethane (DCM). The increased sensing performance of the P3HT:TPFB blend originated from the chemical interaction between the Lewis acid and base.

## 2. Materials and Methods 

Regioregular poly(3-hexylthiophene-2,5-diyl) (P3HT) with a regioregularity of 91–94% and molecular weight of 50–70 kg/mol was purchased from Rieke Metals, Inc (Lincoln, NB, USA). Tris(pentafluorophenyl)borane (TPFB) (purity >98.0%) was purchased from TCI (Tokyo, Japan). Chloroform, dichloromethane, acetone, and octadecyltrichlorosilane (ODTS) were purchased from Sigma-Aldrich (St. Louis, MO, USA). A one-normality ammonium hydroxide solution (1 N NH_4_OH) was purchased from Alfa Aesar (Ward Hill, MA, USA). Methanol was purchased from Daejung Chemicals & Metals (Gyeonggi-do, Korea).

### 2.1. Fabrication of Thin Polymer Films and Polymer Devices

We used a silicon wafer with a 200-nm thermal oxide layer as a substrate. The wafer was sized to 1.7 cm by 1.7 cm and cleaned by using a dust blower (N_2_ gas). This was then washed by sequential ultrasonication in deionized water, acetone, and isopropyl alcohol (for 20 min in each of them) and dried by using a nitrogen-gas blower. The polished side of the wafer was treated with oxygen plasma for 15 min, and then chemically modified with ODTS by immersing the substrate into an ODTS solution (15 µL of ODTS in 12 mL of toluene) for 15 min. The wafer was then ultrasonicated in toluene for 2.5 h. Blend solutions of P3HT and TPFB were prepared in chloroform with a concentration of 10 mg/mL and different solid weight ratios (P3HT:TPFB = 10:0, 9:1, 8:2, and 6:4). The blend solution was spun on the substrate at 3 krpm for 60 s in a glovebox to deposit the polymer film. The film was thermally annealed at 120 °C for 30 min in the dry box to remove the residual solvent and enhance the molecular organization. Gold electrodes (50 nm) were thermally deposited onto the film through a patterned shadow mask to complete the bottom-gate/top-contact transistor fabrications. The length (L) of the device channel was 150 µm, while the channel width (W) was 1.5 mm. 

### 2.2. Characterizations

Ultraviolet–visible (UV-vis) absorption spectra of both solutions and thin films were acquired by using a V-770 spectrophotometer (Jasco, Inc., Easton, MD, USA). The thickness and surface morphologies of the thin films were investigated using an atomic force microscope (AFM; XE-100, Park Systems, Suwon, Korea). The OFETs and sensor devices were placed in an environment-controlled air-tight chamber and characterized by using Keithley 2634B (Tektronix, Beaverton, OR, USA) and HP4156A (Keysight Technologies, Santa Rosa, CA, USA) semiconductor parameter analyzers. The field-effect mobility (*μ*) and threshold voltage (*V*_T_) of the devices were calculated by using the curves of saturation regime (V_d_ = −100 V) and equation: I_d_ = (WC_i_/2L)µ(V_g_ − V_T_)^2^, where I_d_ is the drain current, C_i_ is the capacitance per unit area of dielectric (17.3 nF/cm^2^), and V_g_ is the gate voltage. As an ammonia source, a 1-N ammonia solution was vaporized to obtain the target concentration (ppm) in the chamber. The carrier gas was nitrogen. The chemical response characteristics were quantified based on the current changes upon analyte exposure. 

## 3. Results

**Figure 1:**
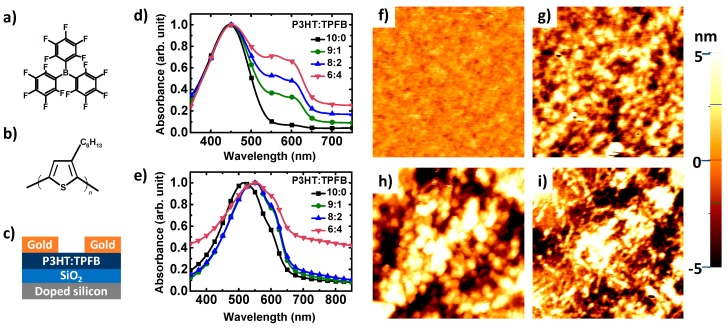
(**a**,**b**) Chemical structures of (**a**) tris(pentafluorophenyl)borane (TPFB) and (**b**) regioregular poly(3-hexylthiophene) (P3HT). (**c**) Schematic of the organic transistor-based sensor. (**d**,**e**) UV-vis absorption spectra of the blends: (**d**) in solutions (5 mg/L in chloroform) and (**e**) in thin films with different P3HT:TPFB ratios. (**f**–**i**) AFM surface topographic images of thin films: (**f**) pure P3HT, (**g**) P3HT:TPFB (9:1), (**h**) P3HT:TPFB (8:2), and (**i**) P3HT:TPFB (6:4). The image size is 2 µm by 2 µm.

The chemical structures of the dopant (TPFB) and semiconducting polymer (P3HT) are depicted in Figure 1a,b. As both components are well soluble in common organic solvents, e.g., chloroform, a thin film of the P3HT:TPFB blend can be easily deposited onto a substrate, which enables the use of the OFETs as a platform to investigate the electrical properties and sensor performance (Figure 1c). TPFB, which has a highly electron-deficient unit, changes the density of positive charge carriers (i.e., holes) in organic and polymer semiconductors by p-type doping [30,31,32]. Therefore, a higher electrical current is expected through the combination of the TPFB dopant with a p-type polymer. The film thickness was governed mainly by the composition of the P3HT solution. The films fabricated by using the solutions (total 10 mg/mL) with P3HT:TPFB weight ratios of 10:0, 9:1, 8:2, and 6:4, had thickness of 42.3, 30.9, 18.0, and 12.8 nm, respectively. 

Figure 1d,e shows UV–vis absorption spectra of the blends, in both solutions and thin films. The main absorption peak of the solution was observed at 450 nm, which corresponds to pure P3HT. Features emerged at 550–600 nm with the increase in concentration of TPFB in the P3HT:TPFB blend. These peaks likely originated from the formation of agglomerates of P3HT in the presence of TPFB. In the solid state, the main absorption peak of P3HT shifted to 515 nm. In addition to the red shift of the main band, shoulders evolved at 550 and 600 nm, as expected for thin films of P3HT. The changes in absorption bands toward larger wavelengths with the increase in TPFB content signify the containment and exclusive effect of TPFB within the film and solution, in good agreement with a previous report [30]. In the range of 350–750 nm, no additional features were developed upon the TPFB addition. Figure 1f–i shows the surface morphology of thin polymer films imaged by AFM. The surface topography in an area of 2 µm by 2 µm became slightly rougher as the dopant content increased, from 0.53 nm for P3HT to 2.54 nm for P3HT:TPFB (9:1), to 3.36 nm for P3HT:TPFB (8:2), and to 3.52 nm for P3HT:TPFB (6:4). 

**Figure 2:**
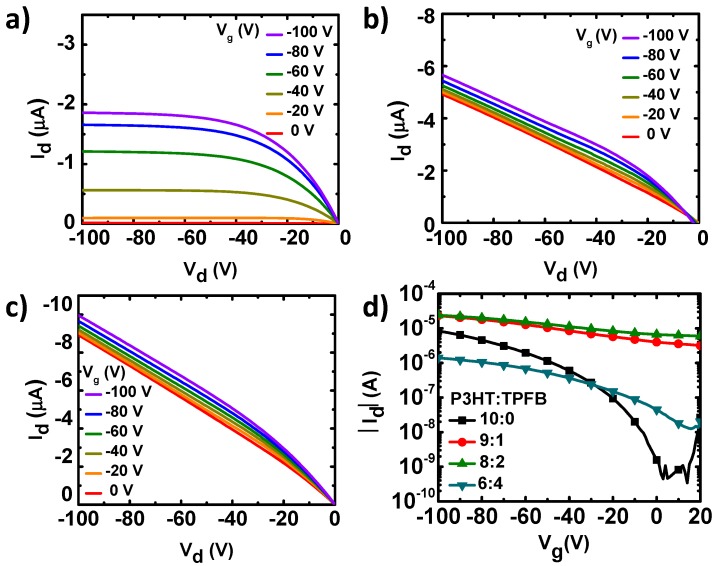
(**a–c**) Output curves of the OFETs with the (**a**) pure P3HT, (**b**) P3HT:TPFB (9:1), and (**c**) P3HT:TPFB (8:2). (**d**) Corresponding transfer curves at *V*_d_ of −100 V.

We fabricated and characterized OFETs based on P3HT and P3HT:TPFB blends (with weight ratios of 9:1, 8:2, and 6:4) as the active charge-transport layers. Figure 2 shows the representative output and transfer curve families of the devices. Owing to the p-type P3HT, we naturally observed a clear modulation of the current by the field-effect hole transport under the negative voltage bias. In the pristine P3HT device, the drain current (I_d_) linearly increased with the drain voltage (*V*_d_) in the linear region, and then saturated at higher drain voltages owing to the pinch-off of the charge accumulation layer. The pure P3HT exhibited a field-effect mobility (*µ*), current on/off ratio (*I*_on_/*I*_off_), and threshold voltage (*V*_T_) of 0.018 (±0.0028) cm^2^ V^−1^ s^−1^, >3.3 × 10^4^, and −23.4 (±1.7) V, respectively, which are in the typical ranges for P3HT devices [22,37]. 

The current–voltage characteristics were, however, different when TPFB was added to P3HT (Figure 2b–d). A linear increase in current was observed for the P3HT:TPFB blends under zero gate bias. The ohmic current originated from the increased charge carrier density by the p-doping of the P3HT:TPFB film [30,31,32]. For the P3HT:TPFB (9:1), the family of output current curves showed the superposition of the ohmic current originated from the doping and field-effect current originated from the negative gate bias (Figure 2b). Owing to the increase in off-state current at a gate voltage of 0 V, the on-to-off current ratio was ~7 for the blend with a ratio of 9:1. The average mobility and threshold voltage were 0.0136 (±0.002) cm^2^ V^−1^ s^−1^ and 44.5 (±12.1) V, respectively, obtained by assuming the gradual channel approximation with a field-effect saturation behavior. The ohmic current was further increased at a higher concentration of TPFB (P3HT:TPFB = 8:2) (Figure 2c). Owing to the mobile charge carriers in the blend with the ratio of 8:2, the on-to-off current ratio was ~4. The generation of excess charge carriers in the active channel has been observed in most doping systems of organic semiconductors [33]. Because of the excess carriers, the addition of TPFB with such a high concentration may not be the best strategy for OFETs. Notably, an excessive amount of dopant has led to a poor charge transport. A large amount of dopant generally increases the charge-carrier density and decreases the mobility. Therefore, the changes of the conductivity depend on the relative degrees of the contributions from the carrier density and mobility. For the blend film with the weight ratio of 6:4, the current level was decreased by a factor of 10–100 compared to those of the blends with less TPFB. The decrease in electrical current was likely caused by the large amount of the non-conducting TPFB component, in combination with the morphological changes, resulting in a large decrease in the charge-carrier mobility. 

After the characterization of the charge-transport properties of the blend films, we compared the ammonia sensing capabilities of the P3HT and P3HT:TPFB films. Considering the formation of a homogenized film, reasonable field-effect mobility, and high current level, the P3HT:TPFB blend film with the ratio of 9:1 was chosen as representative. Our devices were placed in a closed, hand-made chamber with vacuum and gas inlet/outlet-controlled valves. We consecutively vacuumed the chamber and purged it with nitrogen gas several times to eliminate the ambient air. The concentration of the analyte was then controlled by vaporizing a given amount of the chemical under the flow of the purging gas. The concentration of NH_3_ (*C*_NH3_) in the gas chamber was estimated by using the equation; *C*_NH3_ (in ppm) = 10^6^*ρwV*_A_*V*_M_/*MV*_C_, where *ρ* is the density of NH_4_OH (0.91 g/mL), *V*_A_ is the volume of the injected NH_4_OH (in mL), *w* is the mass ratio of NH_3_ in NH_4_OH (for 1-N NH_4_OH, *w* = 0.01932), *M* is the molar mass of NH_3_ (17 g/mol), V_M_ is the molar volume of ideal gas (22.4 L/mol), and *V*_C_ (16 L) is the volume of the gas chamber [38]. 

**Figure 3:**
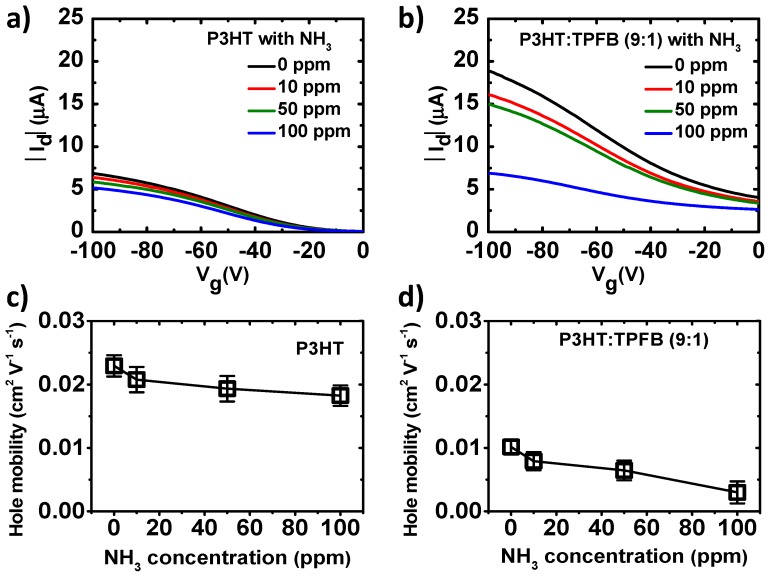
Current–voltage curves and electrical parameters of the P3HT and P3HT:TPFB devices exposed to different NH_3_ concentrations: (**a**) Transfer curves of the OFET with the pristine P3HT with a thickness of 31.3 nm. (**b**) Transfer curves of the OFET with the P3HT:TPFB (9:1) film with a thickness of 30.9 nm. Field-effect mobilities of (**c**) pristine P3HT and (**d**) P3HT:TPFB (9:1) devices. *V*_d_ was fixed at −100 V.

The electrical responses of the polymer films under chemical exposure were then recorded under OFET measurement conditions. The response curves of the pristine P3HT and P3HT:TPFB (9:1) at NH_3_ concentrations of 0, 10, 50, and 100 ppm are shown in Figure 3a,b. To minimize the thickness effects, which may limit the molecular diffusion of the analyte in the solid film, we set the film thickness to ~31 nm. The current level decreased when the P3HT film was exposed to NH_3_ (Figure 3a), similar to the results in the previous reports [9,10,11,12,13,22]. The reduction in current when the device was exposed to more ammonia occurred as the NH_3_ molecules diffused into the polymer layer and lone pairs of electrons of the ammonia reduced the net positive charges (holes) of the film. Consequently, the hole mobility of the p-type polymer was reduced, as shown in Figure 3c. Figure 3b shows the sharper decrease in current in the P3HT:TPFB (9:1) blend compared to that of the pure P3HT. This indicates more sensitive responses of the blend devices even for a wide concentration range of NH_3_, owing to the strong interaction between the boron atom of TPFB and nitrogen atom of ammonia [8,31,32]. The electrophilic borane center binds to the analyte carrying an accessible lone pair of electrons, NH_3_ in our case. The hole mobility of the P3HT:TPFB blend also decreased when the device was exposed to a higher NH_3_ concentration. 

For practical sensing applications, the sensing capabilities of the devices should be quantified. For the evaluation of the sensitivity, we used the percentage current response, %Δ*I*_d_ = 100 × |*I*_d_ − *I*_d0_|/*I*_d0_, where *I*_d_ and *I*_d0_ are the drain currents under and without exposure, respectively [8]. The response of P3HT at *V*_g_ = −100 V was 7.5% at 10 ppm, 12.1% at 50 ppm, and 20.3% at 100 ppm. The responses of P3HT:TPFB (9:1) at the same gate voltage and NH_3_ concentrations were 17.0%, 23.2%, and 65.8%, respectively. The sensitivity was largely increased by the TPFB dopant. The amplifying character of the transistor enabled the sensitivity increase of the blend device. For the P3HT:TPFB (9:1) device at *V*_g_ = 0 V, the percentage responses were lower, 13.6% at 10 ppm, 22.6% at 50 ppm, and 40.4% at 100 ppm (Supplementary Figure S1). The pristine P3HT device at *V*_g_ = 0 V had much lower off-state drain current levels and showed the percentage responses of 14.4%, 18.1%, and 33.6%, respectively. 

**Figure 4:**
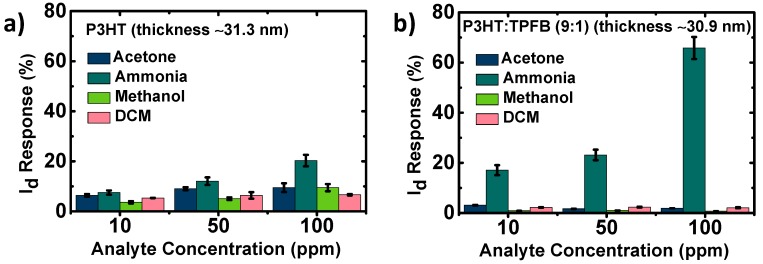
Selectivity of the sensors consisting of the thin polymer films of (**a**) P3HT and (**b**) P3HT:TPFB (9:1). The current was recorded at the gate voltage of −100 V. The film thickness was set to ~31 nm.

Chemical sensors need to selectively detect a certain analyte of interest. Without a sufficient selectivity, the sensor would be disturbed by other competing analytes with similar structures and/or properties, which would affect the correct detection of the target chemical. Therefore, we investigated the current responses of the devices to gas-phase ammonia, acetone, methanol, and DCM, common volatile organic compounds in living environments with relatively simple chemical structures. The pristine P3HT exhibited %Δ*I*_d_ in the range of 3.6–7.5% at 10 ppm of the four analytes without meaningful selectivity, when tested one by one (Figure 4a). This shows the rather low selectivity of the pristine P3HT device toward NH_3_. The responses gradually increased when the device was tested at higher concentrations. The responses were 6.6% for DCM, 9.5% for acetone and methanol, and 20.3% for ammonia at 100 ppm. This shows the limited use of the pristine P3HT device as an ammonia-targeting sensor. On the other hand, the P3HT:TPFB (9:1) device exhibited a considerably more selective response toward ammonia than to the other analytes at all exposure concentrations (Figure 4b). The %Δ*I*_d_ response of the P3HT:TPFB (9:1) device at 10 ppm of NH_3_ analyte was considerably high (17.0%), whereas the responses were 3.1% for acetone, 0.8% for methanol, and 2.2% for DCM. When we tested the device in an environment with four analytes (10 ppm each), the response was 21.4%, which was just 4.4% point higher than the level for 10 ppm NH_3_ (Figure S2). That is, the target analyte is mainly responsible for the sensing response of the P3HT:TPFB (9:1) device and the interference of non-target analytes are negligible. The differences were considerably larger at higher concentrations of the analytes. At 100 ppm, the response to ammonia was 65.8%, whereas those of the other analytes were 0.7–2.1%. 

The impressive sensitivity and selectivity of the combination of P3HT and TPFB originated from the chemical interaction of the blend film with the analyte. TPFB is sufficiently strong to dope the p-type P3HT under normal conditions. However, upon exposure to NH_3_, the higher affinity of the Lewis acid (TPFB) toward the Lewis base (NH_3_) was responsible for the reduction in the P3HT layer, according to the principle of hard acid and hard base [39]. The dedoping of the TPFB-doped P3HT was more significant for the decrease in current level than the reduction in hole mobility in P3HT in the presence of NH_3_, which provided the higher sensitivity of the P3HT:TPFB blend than that of the pristine P3HT upon the exposure. In addition, it is worth noting that, although the interaction between TPFB and NH_3_ is strong, the binding of TPFB and NH_3_ is also reversible. The current level of the P3HT:TPFB blend device could be recovered as the analyte was flushed out by a flow of inert nitrogen gas (Supplementary Figures S3 and S4). It should be also noted that water can be a Lewis base depending on the situation, because water is amphoteric. Therefore, we tested our P3HT:TPFB (9:1) device in the humid environment. The device was insensitive to the H_2_O vapor over a wide range of concentrations from 50 ppm to 5500 ppm, and only exhibited the percent current response of ~1% (Supplementary Figure S5). Hence, P3HT blended with TPFB is promising for applications in solution-processed base-sensing devices. 

## 4. Conclusions

We introduced a selective ammonia-sensing platform based on a solution-processed film of P3HT and p-doping TPFB, and analyzed the direct interaction between the ammonia molecule and P3HT:TPFB blended active layer. The gas sensor with a P3HT:TPFB weight ratio of 9:1 exhibited favorable responses to various NH_3_ concentrations with a significant sensitivity, as shown by the percentage current reduction. Moreover, the P3HT:TPFB blend sensor exhibited a remarkable selectivity with respect to common volatile organic compounds, such as acetone, methanol, and dichloromethane, compared to that of the pristine P3HT device. The performance increase originated from the doping of P3HT by TPFB and dedoping by the stronger hard-acid/hard-base interaction. The use of the proposed system of polymer:dopant blend is an effective approach to the fabrication and modulation of ammonia sensing devices. 

## Supplementary Materials

The following are available online at https://www.mdpi.com/2073-4360/12/1/128/s1, Figure S1: Selectivity of sensors made from thin polymer films: (a) P3HT and (b) P3HT:TPFB=9:1. The current was recorded at the gate voltage of 0 V. The film thickness was controlled to be ~31 nm, Figure S2: Percentage current responses of the P3HT:TPFB (9:1) devices for individual analyte at 10 ppm and the mixture of four analytes (10 ppm each). The current was recorded at the gate voltage of −100 V, Figure S3: Real-time responses of a P3HT:TPFB (9:1) device during multi-cycling of sequential exposure to NH_3_ (10 ppm) and N_2_-gas flush. The current was recorded at the drain and gate voltages of −100 V, Figure S4: Transfer curves of a P3HT:TPFB (9:1) device before NH_3_ exposure, during NH_3_ exposure, and after recovery in N_2_ environment, Figure S5: (a) Transfer curves of a P3HT:TPFB (9:1) device in water vapor. (b) Percentage current responses of a P3HT:TPFB (9:1) device to a various concentration of H_2_O vapor. The current was recorded at the gate voltage of −100 V.
