# Supplementary material for: Selective Ammonia-Sensing Platforms Based on a Solution-Processed Film of Poly(3-Hexylthiophene) and p-Doping Tris(Pentafluorophenyl)Borane
Source: Polymers (Basel). 2020 Jan 5;12(1):128. doi: 10.3390/polym12010128 (PMC7022764; doi:10.3390/polym12010128)
Supplement: Supplementary file 1 [file polymers-12-00128-s001.pdf]

polymers

Supporting Information

Selective Ammonia-Sensing Platforms Based on a Solution-Processed Film of Poly(3-Hexylthiophene) and p-Doping Tris(Pentafluorophenyl)Borane

Alem Araya Meresa and Felix Sunjoo Kim *

School of Chemical Engineering and Materials Science, Chung-Ang University, Seoul 06974, Korea; alemaraya12man@gmail.com

* Correspondence: fskim@cau.ac.kr; Tel.: +82-2-820-5200

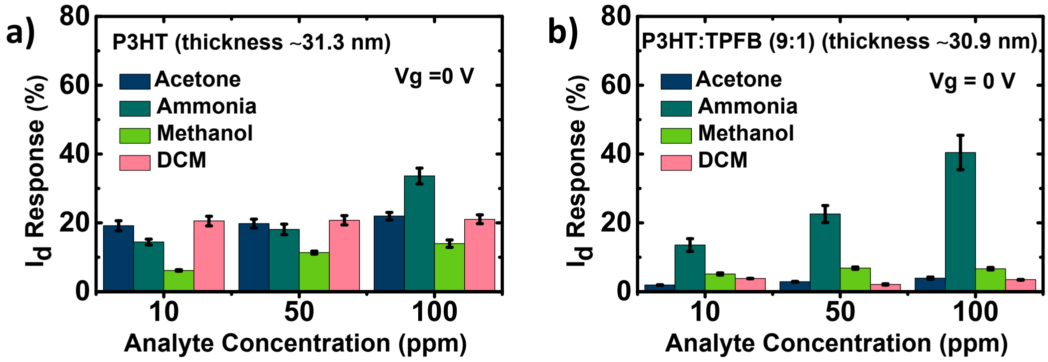

Figure S1. Selectivity of sensors made from thin polymer films: (**a**) P3HT and (**b**) P3HT:TPFB=9:1. The current was recorded at the gate voltage of 0 V. The film thickness was controlled to be ~31 nm.

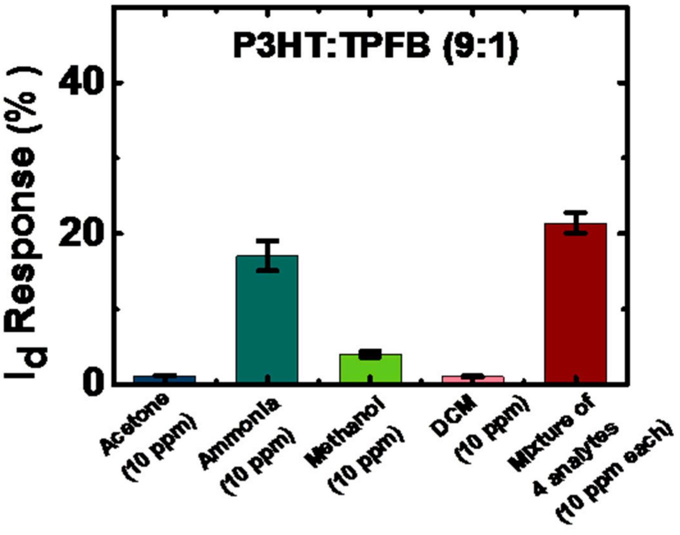

Figure S2. Percentage current responses of the P3HT:TPFB (9:1) devices for individual analyte at 10 ppm and the mixture of four analytes (10 ppm each). The current was recorded at the gate voltage of –100 V.

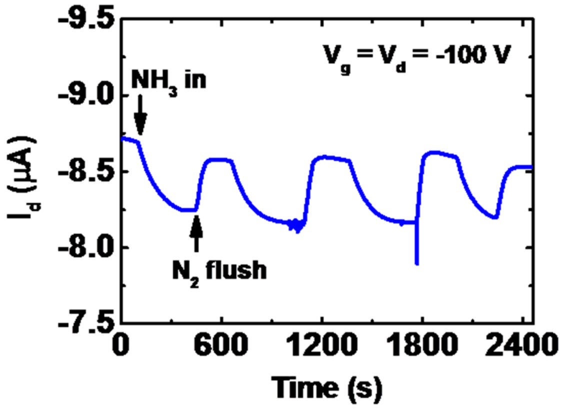

Figure S3. Real-time responses of a P3HT:TPFB (9:1) device during multi-cycling of sequential exposure to NH_3 (10 ppm) and N_2-gas flush. The current was recorded at the drain and gate voltages of −100 V.

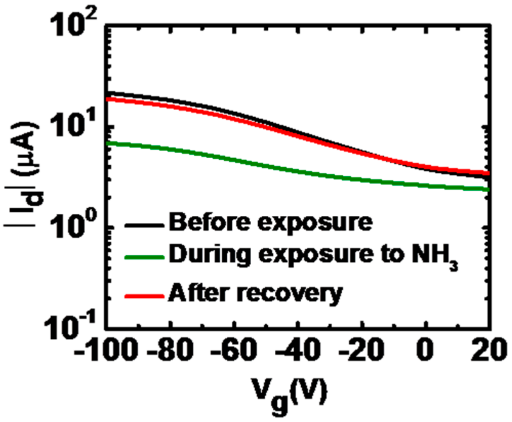

Figure S4. Transfer curves of a P3HT:TPFB (9:1) device before NH_3 exposure, during NH_3 exposure, and after recovery in N_2 environment.

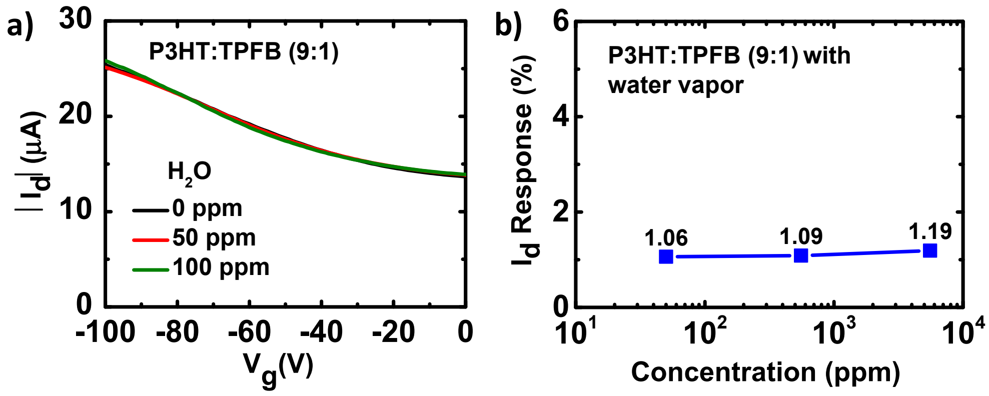

Figure S5. (**a**) Transfer curves of a P3HT:TPFB (9:1) device in water vapor. (**b**) Percentage current responses of a P3HT:TPFB (9:1) device to a various concentration of H_2O vapor. The current was recorded at the gate voltage of −100 V.

© 2020 by the authors. Licensee MDPI, Basel, Switzerland. This article is an open access article distributed under the terms and conditions of the Creative Commons Attribution (CC BY) license (http://creativecommons.org/licenses/by/4.0/).
